# Dietary cholesterol increases body levels of oral administered vitamin D_3_ in mice

**DOI:** 10.1017/jns.2024.32

**Published:** 2024-09-25

**Authors:** Julia Kühn, Alexandra Schutkowski, Lina-Maria Rayo-Abella, Mikis Kiourtzidis, Anika Nier, Corinna Brandsch, Gabriele I. Stangl

**Affiliations:** Institute of Agricultural and Nutritional Sciences, Martin Luther University Halle-Wittenberg, Halle (Saale) 06120, Germany

**Keywords:** Bile acid, Cholesterol, Mass spectrometry, Mice, Vitamin D

## Abstract

Vitamin D and cholesterol share the same intestinal transporters. Thus, it was hypothesized that dietary cholesterol adversely affects vitamin D uptake. The current studies investigated the influence of cholesterol on the availability of oral vitamin D. First, 42 wild-type mice received a diet with 25 µg/kg labelled vitamin D_3_ (vitamin D_3_-d_3_), supplemented with either 0% (control), 0.2%, 0.4%, 0.6%, 0.8%, 1.0% or 2.0% cholesterol for four weeks to investigate vitamin D uptake. In a second study, 10 wild-type mice received diets containing 0% (control) or 1% cholesterol over four weeks to determine cholesterol-induced changes in bile acids. Finally, we investigated the impact of cholesterol versus bile acids on vitamin D uptake in Caco-2 cells. Surprisingly, dietary cholesterol intake was associated with 40% higher serum levels of vitamin D_3_-d_3_ and 2.3-fold higher vitamin D_3_-d_3_ concentrations in the liver compared to controls. The second study showed that cholesterol intake resulted in higher concentrations of faecal bile acids (control: 3.55 ± 1.71 mg/g dry matter; 1% dietary cholesterol: 8.95 ± 3.69 mg/g dry matter; *P* < 0.05) and changes in the bile acid profile with lower contents of muricholic acids (*P* < 0.1) and higher contents of taurodeoxycholic acid (*P* < 0.01) compared to controls. *In-vitro* analyses revealed that taurocholic acid (*P* < 0.001) but not cholesterol increased the cellular uptake of vitamin D by Caco-2 cells. To conclude, dietary cholesterol seems to improve the bioavailability of oral vitamin D by stimulating the release of bile acids and increasing the hydrophobicity of bile.

## Introduction

Vitamin D deficiency is a global public health problem among all age groups.^(1)^ To prevent or treat vitamin D deficiency, many health authorities have established guidelines on vitamin D intake. The National Academy of Medicine recommends a daily oral intake of 15 µg of vitamin D.^(2)^ However, the response of 25-hydroxyvitamin D (25(OH)D), which is used as a vitamin D status marker, to vitamin D supplementation depends not only on endogenous factors such as genetics,^(3,4)^ age^(5)^ and body fat,^(6)^ but also on dietary compounds such as the type of dietary fatty acids,^(7)^ phytosterols^(8)^ or fungal ergosterol.^(9)^ While multiple studies have investigated the role of vitamin D on cholesterol metabolism in humans, the influence of dietary cholesterol on the bioavailability of oral vitamin D is currently unknown. In 2004, Altmann *et al.* ^(10)^ identified Niemann-Pick C1-like 1 (NPC1L1) as a transmembrane protein that is crucial for the intestinal absorption of dietary and biliary cholesterol. In 2011, Reboul *et al.* observed that the cellular uptake of vitamin D was significantly reduced when Caco-2 cells were incubated with ezetimibe, a specific inhibitor of NPC1L1.^(8)^ Additionally, inhibition of NPC1L1 in mice resulted in markedly lower concentrations of vitamin D in the liver, adipose tissues, skeletal muscle, kidney and heart.^(11)^ These data indicate that cholesterol and vitamin D compete for the same absorption mechanism in the gut. Thus, it is tempting to speculate that dietary cholesterol could impact vitamin D status by modulating the intestinal uptake of vitamin D.

Data from the National Health and Nutrition Examination Survey (NHANES) reported that the mean dietary cholesterol intake of U.S. adults in the 2013–2014 survey cycle was 293 mg/day (348 mg/day for men and 242 mg/day for women).^(12)^ However, the dietary cholesterol intake of a person largely depends on the foods and diets that are consumed because foods of animal origin such as eggs and meat are major sources of cholesterol. Since many individuals depend, at least temporarily, on vitamin D supplementation, the question arises whether foods or diets rich in cholesterol may reduce the efficacy of oral vitamin D to improve vitamin D status. Based on the similar chemical structures of cholesterol and vitamin D and the fact that both molecules share the same intestinal transporter, we hypothesized that the consumption of cholesterol could adversely affect vitamin D uptake and in turn vitamin D status.

## Methods

The experimental protocols of the mouse studies were approved by the animal welfare committee of the Martin Luther University Halle-Wittenberg (approval numbers: H1-4/T3-19, H1-4/T1-15). The experimental protocols followed the established guidelines for the care and handling of laboratory animals^(13)^ and were in accordance with the German animal welfare regulations. The studies adhered to the ARRIVE Guidelines for reporting animal research.

### The impact of dietary cholesterol on vitamin D status in mice

The impact of dietary cholesterol on vitamin D status was first examined in a mouse study. All mice were kept in pairs in Macrolon cages in a room with a constant temperature (22 ± 2°C), light cycle (12-h light, 12-h dark with lamps that did not emit UV light) and relative humidity (50 – 60%). The animals had free access to feed and water.

Forty-two male 6-week-old wild-type mice (C57BL/6N) were purchased from Charles River (Sulzfeld, Germany). Mice were given five days to acclimate to their environment before they were randomly assigned into seven groups of six animals each (initial body weight of 21.6 ± 0.93 g). During the study, mice were fed diets with 25 µg/kg triple-deuterated vitamin D_3_ (vitamin D_3_-d_3_, Sigma-Aldrich, Steinheim, Germany) that contained 0% (control group), 0.2%, 0.4%, 0.6%, 0.8%, 1.0% or 2.0% cholesterol for four weeks. The basal diet contained (per kg) 297 g of starch, 200 g of sucrose, 200 g of casein, 150 g of coconut oil, 50 g of soybean oil, 50 g of a vitamin and mineral mixture, 50 g of cellulose, and 3 g of DL-methionine. Varying amounts of cholesterol were added to the diet in exchange for starch. Vitamins and minerals were added to the diet according to the recommendations of the National Research Council.^(14)^

After four weeks of treatment, the mice were deprived of feed for four hours, anaesthetised and decapitated. Feed withdrawal four hours before sampling and dissection of mice were carried out during the light phase of the light-dark cycle, starting between 6:00 am and 10:00 am. The blood samples were taken and collected in tubes (Sarstedt, Nümbrecht, Germany) to obtain serum. Additionally, intestinal mucosa, livers, kidneys and retroperitoneal adipose tissues were harvested. All samples were immediately snap-frozen in liquid nitrogen and stored at -80°C until analyses.

### The effects of dietary cholesterol on bile acids in mice

To investigate the impact of dietary cholesterol on bile acids, which in turn can influence the digestibility and uptake of fat-soluble nutrients, a second study with mice was conducted. The care and handling of the mice were in line with the protocol described above.

Ten male 4-week-old wild-type mice (C57BL/6N; Charles River) with an initial body weight of 14.3 ± 1.49 g were randomly allotted to two groups (n = 5). The mice were fed diets with 25 µg/kg vitamin D_3_ (Sigma-Aldrich) that contained either 0% (control group) or 1.0% cholesterol. The basal diet contained (per kg) 397 g of starch, 200 g of sucrose, 200 g of casein, 100 g of lard, 50 g of a vitamin and mineral mixture, 50 g of cellulose, and 3 g of DL-methionine. Cholesterol was added to the diet in exchange for starch. Vitamins and minerals were added to the diet according to recommendations of the National Research Council.^(14)^

After four weeks of treatment, the mice were deprived of feed for four hours, anaesthetized and decapitated in accordance to mouse study 1 described above. The intestinal mucosa samples were harvested to analyse the intracellular vitamin D concentration, bile was obtained from the gallbladder, and faeces was collected from the rectum to quantify bile acids. The samples were immediately snap-frozen in liquid nitrogen and stored at -80°C until analyses.

### Cell culture study on the effects of cholesterol and bile acids on vitamin D uptake

To elucidate the impact of cholesterol versus bile acids on the cellular uptake of vitamin D_3_, a cell culture study using human colorectal adenocarcinoma Caco-2 cells (ACC 169, German Collection of Microorganisms and Cell Cultures, Braunschweig, Germany) was conducted. The Caco-2 cells, which formed monolayers, were cultivated in Minimal Essential Medium, GlutaMAX® (MEM), supplemented with 1% non-essential amino acids (NEAAs), 10% foetal bovine serum (FBS) and 0.5% gentamycin (all from Gibco, Life Technologies GmbH, Darmstadt, Germany) at 37°C in a humidified atmosphere (95% air and 5% CO_2_). For intracellular vitamin D_3_ analysis, cells were seeded in dishes (diameter: 3.5 cm) at a density of 0.8 × 10^6^ cells per dish. For relative mRNA expression analysis, cells were seeded in 24-well plates at a density of 0.15 × 10^6^ cells per well. The seeded cells were cultured for seven days, to differentiate them into small intestinal epithelial-like cells.^(15,16)^ Eighteen hours prior to incubation, the medium was replaced by FBS-free MEM supplemented with 1% NEAAs. For uptake experiments, cells were incubated in FBS-free MEM with 1 µM vitamin D_3_ for 60 min at 37°C. To this end, vitamin D_3_ was incorporated in micelles as described by Reboul *et al.* ^(8,17)^ All micelles consisted of 0.04 mM L-α-phosphatidylcholine, 0.5 mM oleic acid, 0.3 mM monoolein, 0.16 mM 1-α-lysophosphatidylcholine and 1 µM vitamin D_3_ (all from Sigma-Aldrich). Then, the cells were treated in a two-factorial design with taurocholic acid (1 mM vs. 5 mM) and cholesterol (0 vs. 100 µM cholesterol) (all from Sigma-Aldrich). Taurocholic acid and cholesterol were added to the micellular components. For preparation of the micelles, appropriate volumes of lipids and vitamin D_3_ stock solutions in absolute ethanol were transferred to a glass tube. The solvent was evaporated under nitrogen, and the dried residues were dissolved in MEM containing taurocholic acid. All micelle components were vigorously mixed in a sonication bath at room temperature for 5 min. The viability of the treated cells was assessed by the 3-(4,5-dimethylthiazol-2-yl)-2,5-diphenyltetrazolium bromide (MTT) test. None of the incubation conditions affected cell viability. After the treatments, the cells were washed twice with ice-cold phosphate-buffered saline (PBS), harvested with a cell scraper and centrifuged. The cell pellets were stored at -20°C until further analysis. Protein concentrations of the cell pellets were determined by the Bradford assay.^(18)^ The experiment was independently repeated three times. Analyses from each experiment were run in duplicate (gene expression) or triplicate (vitamin D_3_).

### Analysis of vitamin D metabolites in plasma, tissues and cells

The concentrations of vitamin D_3_, vitamin D_3_-d_3_ and triple-deuterated 25-hydroxyvitamin D_3_ (25(OH)D_3_-d_3_) were measured by liquid chromatography-tandem mass spectrometry (LC-MS/MS) as recently described.^(11)^ In brief, sevenfold deuterated vitamin D_3_ (Toronto Research Chemicals, Inc., Toronto, Canada) and sixfold deuterated 25(OH)D_3_ (Chemaphor Chemical Services, Ottawa, Canada) were added to the samples as internal standards. Subsequently, the samples were saponified with potassium hydroxide, extracted with n-hexane and washed with ultrapure water. Tissue samples were further purified by normal-phase HPLC (1100 Series, Agilent Technologies, Waldbronn, Germany). All types of samples were subjected to derivatization with 4-phenyl-1,2,4-triazoline-3,5-dione (PTAD; Sigma-Aldrich) and analysed by LC-MS/MS (1260 Infinity Series, Agilent Technologies; QTRAP 5500, SCIEX, Darmstadt, Germany) with positive electrospray ionization. For quantification of vitamin D_3_ and vitamin D_3_-d_3_, a Hypersil ODS C18 column (120 A, 5 μm, 150 × 2.0 mm^2^; VDS Optilab, Berlin, Germany) was used, and for quantification of 25(OH)D_3_-d_3,_ a Poroshell C18 column (120 A, 2.7 μm, 50 × 4.6 mm^2^; Agilent Technologies) was used. Quantifier mass transitions of the PTAD adducts were vitamin D_3_ 560 > 298, vitamin D_3_-d_3_ 563 > 301, sevenfold deuterated vitamin D_3_ 567 > 298, 25(OH)D_3_-d_3_ 579 > 301, and sixfold deuterated 25(OH)D_3_ 582 > 298. Calibration curves were constructed with standard solutions for vitamin D_3_-d_3_ and 25(OH)D_3_-d_3_ (both from Sigma-Aldrich) by plotting the ratio of the analyte peak area to the internal standard peak area versus the concentration of the analytes.

### Analysis of triglycerides in liver

Liver samples were prepared as described elsewhere^(19)^ and the triglyceride concentration of the extracts was quantified using an enzymatic reagent kit according to the manufacturer’s manual (DiaSys Diagnostic Systems GmbH, Holzheim, Germany).

### Analysis of the relative mRNA expression of genes involved in vitamin D uptake and metabolism

Relative mRNA expression was analysed by real-time RT-PCR as described previously.^(19)^ Prior to analysis, total RNA was isolated from tissue samples and Caco-2 cells using peqGOLD TriFast™ (VWR International GmbH, Darmstadt, Germany). The concentration of RNA in the samples was determined at a wavelength of 260 nm with a NanoDrop^TM^ Spectrophotometer (Thermo Fisher Scientific GmbH, Schwerte, Germany). Reverse transcription reactions were performed using M-MLV Reverse Transcriptase (Promega, Madison, WI, USA) to yield cDNA, and real-time RT-PCR was performed with GoTaq® Flexi DNA-Polymerase (Promega) on a Rotorgene 6000 cycler (Corbett Research, Mortlake, Australia) according to a protocol described elsewhere.^(19)^ After each PCR run, melting curve analysis and gel electrophoresis verified the amplification and product size. The relative mRNA expression of the target genes was calculated by the method of Pfaffl.^(20)^ Glyceraldehyde-3-phosphate dehydrogenase *(Gapdh*/*GAPDH)* and ribosomal protein lateral stalk subunit P0 (*Rplp0*/*RPLP0)* were used as the appropriate reference genes. Primers of the target and reference genes are summarised in Table 1.

**Table 1.** Primers of target genes involved in vitamin D uptake and metabolism and appropriate reference genes

	Obtained from	Accession number	Product size [bp]
Mouse primer			
* Npc1l1*	Eurofins MWG Synthesis	NM_207242.2	76
* Cd36*	Eurofins MWG Synthesis	NM_001159558.1	207
* Abcg5*	Eurofins MWG Synthesis	NM_031884.1	77
* Abcg8*	Eurofins MWG Synthesis	NM_026180.2	73
* Cyp27a1*	Sigma-Aldrich	NM_024264.4	126
* Cyp2r1*	Sigma-Aldrich	NM_177382.4	196
* Gapdh* ^*^	Eurofins MWG Synthesis	XM_001473623.1	177
* Rplp0* ^*^	Eurofins MWG Synthesis	NM_007475.5	146
Human primer			
* NPC1L1*	Eurofins MWG Synthesis	NM_001101648.1	164
* CD36*	Eurofins MWG Synthesis	NM_001001548.2	172
* ABCG5*	Eurofins MWG Synthesis	NM_022436.3	234
* ABCG8*	Sigma-Aldrich	NM_001357321.2	199
* SCARB1*	Sigma-Aldrich	NM_016741.2	149
* GAPDH* ^*^	Eurofins MWG Synthesis	NM_002046.3	453
* RPLP0* ^*^	Eurofins MWG Synthesis	NM_001002.3	223

* Reference gene. Abcg5/ABCG5, ATP-binding cassette subfamily G member 5; Abcg8/ABCG8, ATP-binding cassette subfamily G member 8; Cd36/CD36, CD36 molecule; Cyp2r1, vitamin D 25-hydroxylase; Cyp27a1, sterol 27-hydroxylase; Gapdh/GAPDH, glyceraldehyde-3-phosphate dehydrogenase; Npc1l1/NPC1L1, Niemann-Pick C1-like 1; Rplp0/RPLP0, ribosomal protein lateral stalk subunit P0; SCARB1, scavenger receptor class B member 1.

### Analysis of faecal bile acids

The concentration of bile acids was determined in the faeces of mice obtained from the rectum by MS-Omics (Vedbaek, Netherlands). The freeze-dried samples were extracted with methanol, transferred to centrifuge tube filters and centrifuged for purification. The filtrate was then subjected to a Thermo Scientific Vanquish LC coupled to Thermo Q Exactive HF mass spectrometer for bile acid analysis. An electrospray ionisation interface was used as the ionisation source. The system was operated in negative ionisation mode. Chromatographic separation of the bile acids was carried out on a Waters Acquity HSS T3 1.8 μm 2.1 × 150 mm. The column was thermostatted at 30°C. The mobile phases consisted of (A) ammonium acetate (10 mmol/l) and (B) methanol/acetonitrile (1/1, v/v). Bile acids were eluted by increasing the concentration of B in A from 45 to 100% over 16 min. The flow rate was 0.3 ml/min. Peak areas were extracted using TraceFinder 4.1 (Thermo Fisher Scientific, Waltham, USA). Identification of the compounds was based on the accurate mass and retention time of the authentic standards.

### Statistical analysis

Data are expressed as the mean ± standard deviation. Statistical analyses were performed using SPSS version 25.0 (IBM, Armonk, NY, USA). Data obtained from the first mouse study were tested for normal distribution (Shapiro-Wilk test) and homoscedasticity (Levene’s test). Treatment effects were identified by one-way ANOVA for normally distributed data. In the case of significant treatment effects, an appropriate post hoc group comparison was performed (Tukey’s test for equal variances or Games-Howell for unequal variances). For parameters that were not normally distributed, the effects of treatment were analysed by the non-parametric Kruskal-Wallis test. Individual group comparisons were performed by the Mann-Whitney U test with Bonferroni’s correction. A correlation analysis of liver triglyceride concentration and liver vitamin D_3_-d_3_ concentration was performed by Spearman correlation as a non-parametric measure of correlation. If not otherwise stated, all mice (N = 42) were included in the analysis. For the second mouse study, data were subjected to Student’s t-test in cases of normal distribution or the non-parametric Mann-Whitney U test. All mice (N = 10) were included in the analysis. Data from the cell culture experiment were analysed by two-way ANOVA including the factors of taurocholic acid treatment, cholesterol treatment and their interaction (taurocholic acid × cholesterol). *P* < 0.05 was designated as a significant difference and *P* < 0.1 as a trend toward significance.

## Results

### The impact of dietary cholesterol on vitamin D status

#### Body weight, liver weight, liver triglycerides and feed intake

The final body weights, liver weights and the liver:body weight ratios as well as the concentrations of triglycerides in the liver of the mice were not differentially affected by the dietary treatments (Table 2). Also, daily feed intake (assessed from two mice per cage) did not differ between the groups (Table 2).

**Table 2.** Body weight, liver weight, relative liver weight, liver triglyceride and feed intake of mice fed different doses of cholesterol

	Dietary cholesterol content							Effect of treatment
	0.0 %	0.2 %	0.4 %	0.6 %	0.8 %	1.0 %	2.0 %	*P* value
Body weight [g]	26.1 ± 1.89	25.7 ± 0.90	24.5 ± 1.54	26.2 ± 2.35	24.3 ± 2.35	25.8 ± 1.42	25.4 ± 1.63	Ns
Liver weight [g]	1.08 ± 0.10	1.04 ± 0.14	0.98 ± 0.20	1.19 ± 0.18	1.00 ± 1.18	1.03 ± 0.15	1.07 ± 0.10	Ns
Liver:body weight ratio [%]	4.14 ± 0.22	4.04 ± 0.53	4.00 ± 0.70	4.53 ± 0.37	4.12 ± 0.37	4.02 ± 0.57	4.22 ± 0.21	Ns
Liver triglyceride [mg/g]	37.9 ± 12.3	30.1 ± 6.16	50.0 ± 15.0	47.3 ± 24.9	52.8 ± 27.6	32.3 ± 6.20	37.6 ± 3.30	Ns
Feed intake [g/d]^a^	4.79 ± 0.18	4.95 ± 0.03	4.18 ± 0.18	4.99 ± 0.02	4.79 ± 0.07	5.35 ± 1.63	4.95 ± 0.28	Ns

Ns, not significant.

a Feed intake was assessed from two mice per cage. Data are expressed as the means ± standard deviation. Treatment effects were analysed by one-way ANOVA or Kruskal-Wallis test.

#### Dietary cholesterol increases vitamin D_3_-d_3_ in serum and tissues

**Fig. 1.**
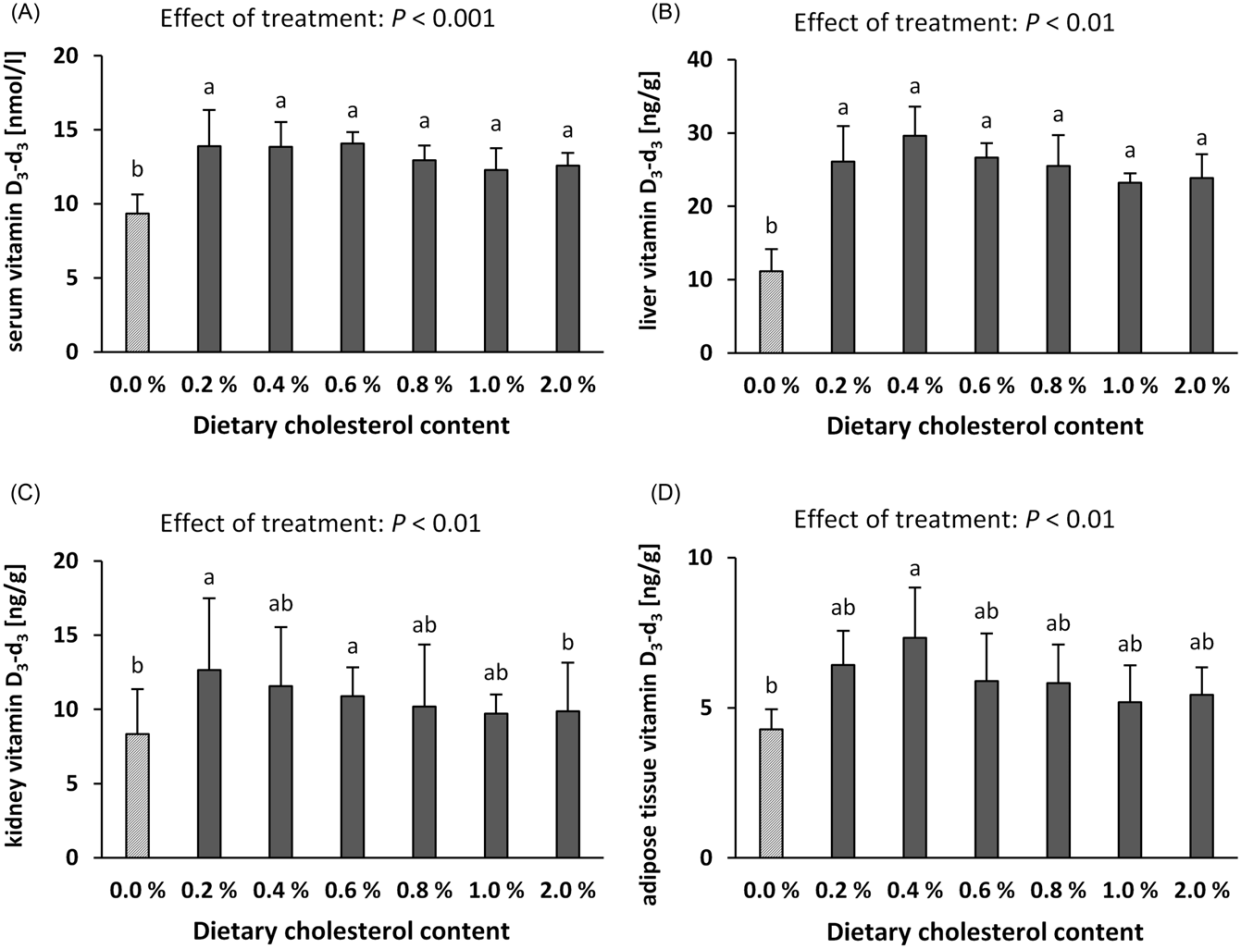
Concentration of vitamin D_3_-d_3_ in the serum (a), livers (b), kidneys (c) and adipose tissues (D) of mice fed different doses of cholesterol. Data are expressed as the means ± standard deviation. Treatment effects were identified by one-way ANOVA or the Kruskal-Wallis test. ^a,b^ Different letters indicate differences between groups (multiple group comparison, *P* < 0.05). Vitamin D_3_-d_3_, triple-deuterated vitamin D_3_.

**Fig. 2.**
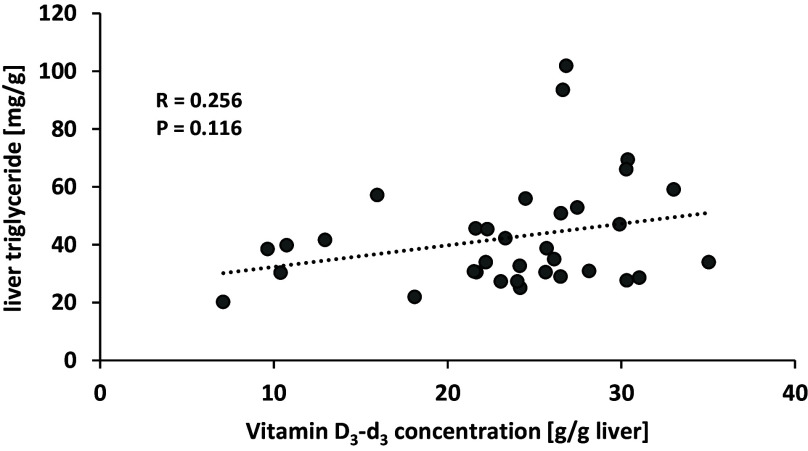
Correlation between concentrations of liver triglycerides and liver vitamin D_3_-d_3_ of mice that were fed different doses of cholesterol. No correlation was identified by Spearman correlation, N = 39.

To assess the effects of dietary cholesterol on vitamin D uptake and vitamin D status, the amounts of deuterated vitamin D metabolites were determined in serum, liver, kidney and adipose tissue. The data showed that the serum and tissue concentrations of vitamin D_3_-d_3_ were significantly influenced by cholesterol in the diet (Fig. 1). Interestingly, the mice fed cholesterol-containing diets had higher serum and tissue concentrations of vitamin D_3_-d_3_ than mice fed the cholesterol-free diet, although no clear dose-response relationship between dietary cholesterol and levels of vitamin D_3_-d_3_ was observed. The increase in vitamin D_3_-d_3_ in response to dietary cholesterol was most pronounced in the serum and liver (Fig. 1a and b). To test whether the higher vitamin D_3_-d_3_ levels in the cholesterol-fed mice were associated with higher triglyceride concentrations in the liver, a correlation between both parameters was performed. As depicted in Fig. 2, no significant correlation was observed between liver triglycerides and the liver vitamin D_3_-d_3_ concentration. Due to the high variance, the increase in vitamin D in the kidney and adipose tissue of mice fed the cholesterol diets did not consistently reach statistical significance (Fig. 1c and d). The concentrations of 25(OH)D_3_-d_3_ in the serum (0% cholesterol group: 59.1 ± 8.89 nmol/l; 0.2%: 57.5 ± 6.94 nmol/l; 0.4%: 54.1 ± 11.7 nmol/l; 0.6%: 68.5 ± 10.6 nmol/l; 0.8%: 57.9 ± 9.56 nmol/l; 1.0%: 64.7 ± 8.56 nmol/l; 2.0%: 61.8 ± 6.80 nmol/l) and kidneys (0% cholesterol group: 2.90 ± 0.23 ng/g; 0.2%: 3.30 ± 0.21 ng/g; 0.4%: 3.39 ± 0.41 ng/g; 0.6%: 3.57 ± 0.49 ng/g; 0.8%: 3.15 ± 0.68 ng/g; 1.0%: 3.52 ± 0.71 ng/g; 2.0%: 3.52 ± 0.55 ng/g) were not affected by dietary cholesterol. The levels of 25(OH)D_3_-d_3_ in the liver and adipose tissue were lower than the limit of quantification (6 ng/g and 2 ng/g, respectively).

#### Dietary cholesterol did not modify the mRNA expression of genes involved in vitamin D uptake and metabolism

To determine whether the increased serum and tissue levels of vitamin D were caused by a higher abundance of uptake transporters, we analysed the mRNA expression of intestinal vitamin D transporters. These data did not indicate any consistent effect of dietary cholesterol on the mRNA expression of transporters involved in vitamin D uptake (Table 3). In addition, we analysed the mRNA expression of the most important hydroxylases in the liver but found no differences between the groups (Table 3).

**Table 3.** Relative mRNA expression of genes involved in vitamin D_3_ uptake and metabolism in mice fed different doses of cholesterol

	Dietary cholesterol content							Effect of treatment
	0.0%	0.2%	0.4%	0.6%	0.8%	1.0%	2.0%	(*P* value)
Intestinal mucosa								
* Npc1l1*	1.00 ± 0.43	0.89 ± 0.19	0.95 ± 0.29	0.80 ± 0.27	0.85 ± 0.23	0.67 ± 0.09	0.97 ± 0.25	Ns
* Cd36*	1.00 ± 0.34	1.22 ± 0.26	1.35 ± 0.62	1.30 ± 0.61	1.24 ± 0.31	1.13 ± 0.53	0.80 ± 0.16	Ns
* Abcg5*	1.00 ± 0.26^ab^	1.16 ± 0.28^ab^	1.46 ± 0.37^ab^	1.60 ± 0.64^a^	1.34 ± 0.26^ab^	0.78 ± 0.28^b^	1.68 ± 0.33^a^	< 0.01
* Abcg8*	1.00 ± 0.40	1.19 ± 0.51	0.88 ± 0.35	1.43 ± 0.46	0.89 ± 0.25	1.02 ± 0.30	0.74 ± 0.36	Ns
Liver								
* Cyp27a1*	1.00 ± 0.21	1.05 ± 0.32	0.93 ± 0.26	0.74 ± 0.21	0.96 ± 0.26	0.81 ± 0.16	0.99 ± 0.12	Ns
* Cyp2r1*	1.00 ± 0.46	1.05 ± 0.39	0.78 ± 0.35	0.87 ± 0.10	0.73 ± 0.34	0.86 ± 0.41	1.47 ± 0.60	Ns

Abcg5, ATP-binding cassette subfamily G member 5; Abcg8, ATP-binding cassette subfamily G member 8; Cd36, CD36 molecule; Cyp2r1, vitamin D 25-hydroxylase; Cyp27a1, sterol 27-hydroxylase; Npc1l1, Niemann-Pick C1-like 1; ns, not significant.

Data are expressed as the means ± standard deviation. Treatment effects were identified by one-way ANOVA or Kruskal-Wallis test. ^a,b^ Different letters indicate differences between groups (multiple group comparison, *P* < 0.05).

### The impact of cholesterol on intestinal vitamin D and bile acids

To elucidate whether cholesterol feeding is associated with higher intestinal bile acid concentrations and in turn, higher vitamin D concentrations in enterocytes *in vivo*, we conducted a subsequent study. Mice that were fed a vitamin D-adequate diet with 0 or 1% cholesterol over four weeks did not show differences in their final body weights (0% cholesterol: 22.2 ± 0.99 g, 1% cholesterol: 22.4 ± 1.62 g).

**Fig. 3.**
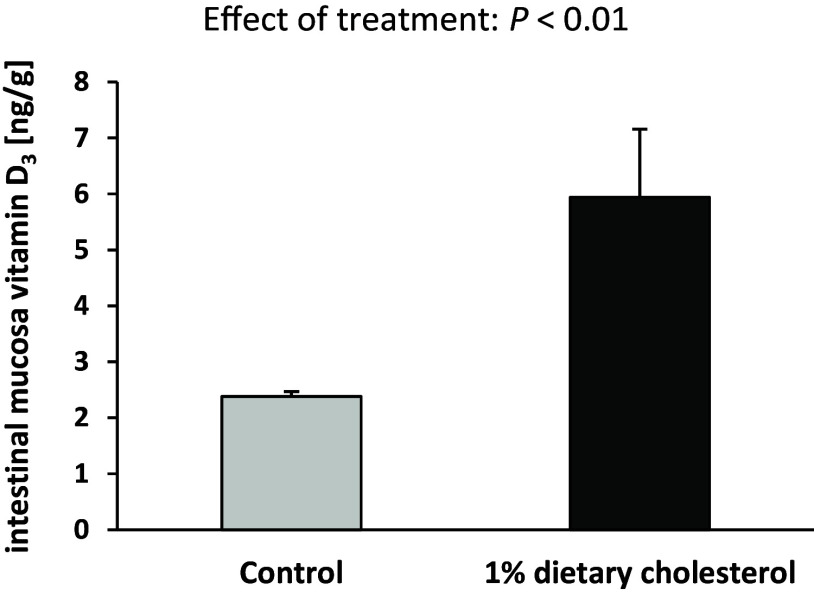
Concentration of vitamin D_3_ in the intestinal mucosa of mice in response to dietary cholesterol supply. Data are expressed as the means ± standard deviation. Treatment effect was identified by Student’s t-test.

Here, we found that the vitamin D_3_ concentration in the intestinal mucosa was significantly higher in mice fed 1% cholesterol than in those fed 0% cholesterol (*P* < 0.01, Fig. 3). In addition, the faecal concentrations of total and secondary bile acids were significantly higher in the mice fed 1% cholesterol than in those fed 0% cholesterol. The primary and tertiary bile acids showed a trend towards higher levels in the cholesterol group (Table 4).

**Table 4.** Faecal bile acid concentration [µg/g dry matter] of mice in response to dietary cholesterol supply

	Control	1% dietary cholesterol	*P* value
Total bile acids	3545 ± 1708	8951 ± 3685	< 0.05
Total primary bile acids	1402 ± 1498	4765 ± 3361	< 0.1
Cholic acid	43.2 ± 36.1	369 ± 324	< 0.1
Chenodeoxycholic acid	3.45 ± 2.24	6.62 ± 5.38	Ns
Glycocholic acid	< LOQ	18.7 ± 33.3	< 0.05
Glycochenodeoxycholic acid	0.95 ± 0.08	1.57 ± 0.89	< 0.05
Taurocholic acid	83.2 ± 149	343 ± 414	Ns
Taurochenodeoxycholic acid	3.89 ± 4.32	8.86 ± 8.17	Ns
α-Muricholic acid	151 ± 151	348 ± 369	Ns
Tauro-α-muricholic acid	35.5 ± 51.4	53.4 ± 43.6	Ns
β-Muricholic acid	725 ± 372	1721 ± 561	< 0.05
Tauro-β-muricholic acid	402 ± 741	1701 ± 2340	Ns
Hyocholic acid	8.51 ± 0.46	26.0 ± 26.7	Ns
Total secondary bile acids	2420 ± 129	4144 ± 1581	< 0.05
Lithocholic acid	17.9 ± 9.65	61.3 ± 14.2	< 0.001
Isolithocholic acid	2.37 ± 1.42	3.01 ± 0.94	Ns
Ketolithocholic acid	7.56 ± 4.22	26.2 ± 17.3	< 0.01
Taurolithocholic acid	0.54 ± 0.19	4.33 ± 3.86	< 0.05
Deoxycholic acid	314 ± 95.9	990 ± 82.5	< 0.05
Hyodeoxycholic acid	21.2 ± 8.17	61.7 ± 11.4	< 0.001
Glycodeoxycholic acid	1.08 ± 0.02	1.82 ± 0.98	< 0.05
Taurodeoxycholic acid	2.35 ± 4.60	61.6 ± 67.5	< 0.1
ω-Muricholic acid	1943 ± 183	2711 ± 1436	Ns
Total tertiary bile acids	13.8 ± 13.5	42.0 ± 24.1	< 0.1
Ursodeoxycholic acid	7.63 ± 5.06	22.2 ± 15.5	< 0.1
Glycoursodeoxycholic acid	1.46 ± 0.02	1.67 ± 0.27	Ns
Tauroursodeoxycholic acid	6.09 ± 9.32	18.2 ± 22.3	Ns

Ns, not significant.

Data are expressed as the means ± standard deviation. Differences between the two groups were identified by Student’s t-test or Mann-Whitney U test.

**Fig. 4.**
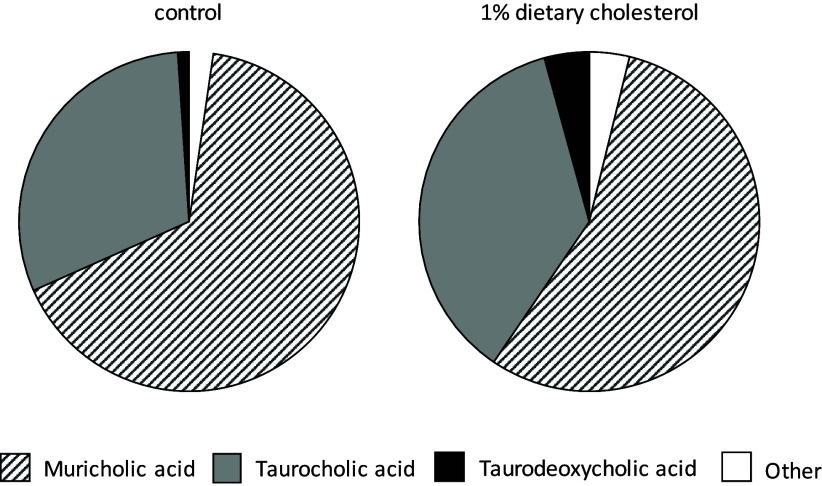
Major bile acids (weight % of the total) in the bile of mice in response to dietary cholesterol supply. Data are expressed as the means. Taurodeoxycholic acid was significantly different (*P* < 0.01), and muricholic acids showed a trend towards significance (*P* < 0.1) in response to dietary cholesterol (Student’s t-test).

The three major types of bile acids found in bile obtained from the gallbladder were muricholic acids, taurocholic acid and taurodeoxycholic acid. Dietary cholesterol intake was associated with a change in the bile acid composition in the bile (Fig. 4). The bile acid profiles of the mice fed 1% cholesterol were characterized by a higher proportion of hydrophobic taurodeoxycholic acid (*P* < 0.01) and a trend towards less hydrophilic muricholic acids (*P* < 0.1, Fig. 4).

### Taurocholic acid but not cholesterol increases the uptake of vitamin D in Caco-2 cells

**Fig. 5.**
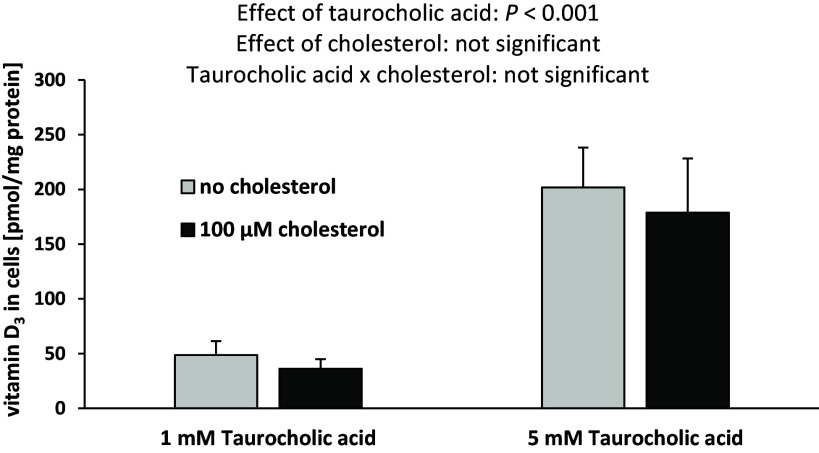
Concentration of vitamin D_3_ in Caco-2 cells in response to micellar cholesterol and taurocholic acid content. Data are expressed as the means ± standard deviation. Treatment effects were identified by two-way ANOVA.

To investigate whether cholesterol can directly improve the cellular uptake of vitamin D or indirectly by the changes in bile acids, we treated Caco-2 cells with micelles differing in cholesterol and taurocholic acid concentrations. Treatment of cells with 100 µM versus 0 µM cholesterol did not change the intracellular vitamin D_3_ concentration, suggesting that cholesterol is not capable of modifying vitamin D uptake in Caco-2 cells per se (Fig. 5). In contrast, the intracellular vitamin D_3_ concentration was approximately fourfold higher after incubation with 5 mM taurocholic acid than with 1 mM taurocholic acid, indicating a strong effect of taurocholic acid in improving vitamin D_3_ uptake into intestinal cells (Fig. 5). Neither cholesterol nor taurocholic acid altered the relative mRNA expression of vitamin D transporters in Caco-2 cells (Table 5).

**Table 5.** Relative mRNA expression of genes involved in vitamin D uptake in Caco-2 cells

	1 mM TCA		5 mM TCA		*P* value		
	No chol	100 µM chol	No chol	100 µM chol	TCA	chol	TCA×chol
*NPC1L1*	1.00 ± 0.14	0.98 ± 0.18	0.87 ± 0.05	1.03 ± 0.28	Ns	Ns	Ns
*CD36*	1.00 ± 0.46	1.29 ± 0.26	1.20 ± 0.32	0.99 ± 0.44	Ns	Ns	Ns
*ABCG5*	1.00 ± 0.11	1.03 ± 0.08	1.61 ± 0.94	1.08 ± 0.22	Ns	Ns	Ns
*ABCG8*	1.00 ± 0.38	1.05 ± 0.43	0.99 ± 0.23	0.94 ± 0.49	Ns	Ns	Ns
*SCARB1*	1.00 ± 0.22	1.44 ± 0.11	1.03 ± 0.08	1.15 ± 0.44	Ns	Ns	Ns

ABCG5, ATP-binding cassette subfamily G member 5; ABCG8, ATP-binding cassette subfamily G member 8; Chol, cholesterol; CD36, CD36 molecule; NPC1L1, Niemann-Pick C1-like 1; ns, not significant; SCARB1, scavenger receptor class B member 1; TCA, taurocholic acid.

Data are expressed as the means ± standard deviation. Treatment effects were identified by two-way ANOVA.

## Discussion

In the current study, we tested the hypothesis that cholesterol could affect the intestinal uptake of oral vitamin D and in turn vitamin D status. Surprisingly, we found significant increases in vitamin D in the serum and livers of mice fed cholesterol, indicating that cholesterol is able to increase the uptake of orally administered vitamin D, although 25(OH)D, which is normally used as a biomarker of vitamin D status, remained unaffected. This finding contradicts the hypothesis that cholesterol and vitamin D compete for the same intestinal transporter and that cholesterol, which was in excess compared to vitamin D, may hinder the absorption of vitamin D. NPC1L1 plays a crucial role in the absorption of cholesterol by enterocytes^(10)^ and is important for vitamin D uptake.^(8,11)^ However, the current mRNA data were not indicative that the improvement in vitamin D status that we observed in the cholesterol-fed groups was caused by cholesterol-induced changes in the expression of NPC1L1 and other transporters, such as CD36 molecule (CD36) or ATP-binding cassette subfamily G member 5/8 (ABCG5/8), which have been suggested to be involved in vitamin D absorption.^(8,21)^ Thus, these data were not indicative of any direct effect of cholesterol on vitamin D transporter expression. However, it should be noted that the intestinal uptake of cholesterol and vitamin D involves both transporter-mediated uptake and passive diffusion. It is therefore possible that the higher vitamin D_3_-d_3_ levels in cholesterol-fed mice resulted from higher passive diffusion.

Cholesterol serves as a precursor for bile acid synthesis and stimulates the formation of bile acids in the liver.^(22,23)^ This explains why mice fed a high-cholesterol diet had substantially higher concentrations of bile acids in their faeces than mice fed no cholesterol. Based on these data, we hypothesised that cholesterol could enhance vitamin D uptake indirectly by increasing the formation and release of bile acids into the gut. This assumption was confirmed in the two subsequent studies that we conducted. First, the concentrations of faecal bile acids and vitamin D in enterocytes were significantly higher in mice fed the cholesterol-containing diet than in mice fed the cholesterol-free diet. Second, analysis of the human Caco-2 cells revealed a substantially higher cellular uptake of vitamin D when micelles contained higher concentrations of taurocholic acid but not when they contained cholesterol. This cell culture study clearly indicates a direct effect of bile acids on vitamin D uptake, but the findings need to be confirmed in further *in vivo* studies.

Bile acids serve as micelle-forming surfactants and facilitate the absorption of lipids and hydrophobic nutrients. Micelles are usually self-assembling structures that are formed from lipid digestion products and bile acids,^(24)^ and their shape, size and composition might determine their efficiency in carrying lipophilic compounds. Thus, we assume that cholesterol could have influenced bile acid formation, release and profile and, in turn, the composition of the micelles and the solubility of vitamin D within micelles. A study revealed that mice deficient in 7α-hydroxylase, an enzyme that plays a key role in the synthesis of bile acids, had low serum vitamin D levels, emphasizing an important role of the classic bile acid pathway in intestinal vitamin D uptake.^(25)^ Moreover, the co-supplementation of vitamin D and cholic acid restored the serum vitamin D levels more efficiently than vitamin D supplementation alone in these mice.^(25)^ Bile acids largely differ in their hydrophobicity, which in turn influences sterol absorption.^(26)^ Supplementation with hydrophobic cholic acid was associated with a higher micellar cholesterol concentration and increased absorption of cholesterol in healthy subjects.^(27)^ In contrast, the administration of hydrophilic muricholic acids resulted in low cholesterol absorption in mice in comparison with the most hydrophobic bile acids (cholic acid and deoxycholic acid).^(26)^ In our mouse study, muricholic acids were the most prominent bile acids found in bile, comprising 65% of the total bile acids in the controls. However, it must be mentioned that muricholic acids belong to a group of bile acids primarily found in mice.^(28)^ Since these bile acids are absent in humans, it is to be expected that the effect of cholesterol on vitamin D uptake in humans differs from that of mice. Feeding cholesterol reduced the content of muricholic acids to 55%. Conversely, the percentage of hydrophobic taurodeoxycholic acid increased in response to a cholesterol-containing diet. Thus, we speculate that the improved uptake of oral vitamin D can also be attributed to a more hydrophobic bile acid pool after dietary cholesterol intake.

Notably, our study did not show a clear dose-response relationship between dietary cholesterol intake and vitamin D_3_-d_3_ concentration in serum and tissue. The maximum levels of vitamin D_3_-d_3_ in serum and tissue were already reached at the lowest dietary cholesterol concentration. Dietary cholesterol administration usually predisposes mice to develop liver steatosis which is associated with the accumulation of lipid droplets in the liver.^(29)^ Thus, it is tempted to speculate that higher levels of liver lipids may explain the higher levels of vitamin D_3_-d_3_ in the cholesterol-fed mice. However, data from the current study are not indicative of higher liver triglyceride levels in the cholesterol-fed mice and there was no correlation between triglycerides and vitamin D_3_-d_3_ in the liver. Instead, we assume that the lowest dose of cholesterol already had the maximum effect on the formation of bile acids or that higher doses of cholesterol had partly displaced vitamin D from the micelles, as has been observed for phytosterols.^(30)^ This would have to be investigated in further studies.

### Conclusions

To conclude, the data demonstrate that dietary cholesterol increases body concentrations of vitamin D, shown by the significant rises of vitamin D in the serum and livers of cholesterol-fed mice. However, these findings are not indicative of a direct cholesterol effect on the absorption of vitamin D but indicate that ingested cholesterol might stimulate the formation and release of bile acids, which in turn increases the micellar solubility of vitamin D and its intestinal uptake.

## Data Availability

The datasets generated and analysed during the current study are available from the corresponding author on reasonable request.

## Abbreviations

**Abcg5/ABCG5:** ATP-binding cassette subfamily G member 5; **Abcg8/ABCG8:** ATP-binding cassette subfamily G member 8; **Chol:** cholesterol; **Cd36/CD36:** CD36 molecule; **Cyp2r1:** vitamin D 25-hydroxylase; **Cyp27a1:** sterol 27-hydroxylase; **FBS:** foetal bovine serum; **Gapdh/GAPDH:** glyceraldehyde-3-phosphate dehydrogenase; **LC-MS/MS:** liquid chromatography-tandem mass spectrometry; **MEM:** minimal essential medium; **NEAAs:** non-essential amino acids; **NHANES:** National Health and Nutrition Examination Survey; **Npc1l1/NPC1L1:** Niemann-Pick C1-like 1; **ns** not significant; **PBS:** phosphate-buffered saline; **PTAD:** 4-phenyl-1,2,4-triazoline-3,5-dione; **Rplp0/RPLP0:** ribosomal protein lateral stalk subunit P0; **SCARB1:** scavenger receptor class B member 1; **TCA:** taurocholic acid; **vitamin D_3_-d_3_:** triple-deuterated vitamin D_3_; **25(OH)D_3_-d_3_:** triple-deuterated 25-hydroxyvitamin D_3_; **25(OH)D:** 25-hydroxyvitamin D
